# Persistent Salmon Patch on the Forehead and Glabellum in a Chinese Adult

**DOI:** 10.1155/2014/139174

**Published:** 2014-05-14

**Authors:** Alexander K. C. Leung, Benjamin Barankin, Kam Lun Hon

**Affiliations:** ^1^Department of Pediatrics, The University of Calgary, The Alberta Children's Hospital, Calgary, AB, Canada T2M 0H5; ^2^Toronto Dermatology Centre, Toronto, ON, Canada M3H 5Y8; ^3^Department of Paediatrics, Chinese University of Hong Kong, Shatin, Hong Kong

## Abstract

Salmon patches are present in approximately 44% of all neonates. The lesions tend to fade with time and those on the glabellum, eyelids, nose, and upper lip are rarely detected after the age of 6. We report a 33-year-old Chinese female with a salmon patch on the forehead and glabellum. To our knowledge, the occurrence of a salmon patch on the forehead and glabellum in adulthood has not been reported. The persistent salmon patch on the face of an adult is benign and not associated with any neurocutaneous syndrome or underlying vascular abnormality. The color of the lesion can be ameliorated with laser therapy if cosmesis is a concern.

## 1. Introduction

Salmon patches, also known as nevus flammeus simplex, are the most common vascular lesions in infancy [1]. Colloquially, the lesions on the forehead and eyelids are known as “angel's kisses” and the ones in the occipital area as “stork bite marks” [1]. Presumably, salmon patches are composed of ectatic dermal capillaries that represent the persistence of fetal circulating patterns in the skin [2]. In the Caucasian population, salmon patches are present in approximately 44% of all neonates [2]. They are much less common in dark-skinned neonates [3]. Both sexes are equally affected [3]. The lesions tend to fade with time and those on the glabellum, eyelids, nose, and upper lip are rarely detected after the age of 6 [1]. We describe a 33-year-old female with a salmon patch on the forehead and glabellum. To our knowledge, the occurrence of a salmon patch on the forehead and glabellum in adulthood has not been reported.

## 2. Case Report

**Figure 1:**
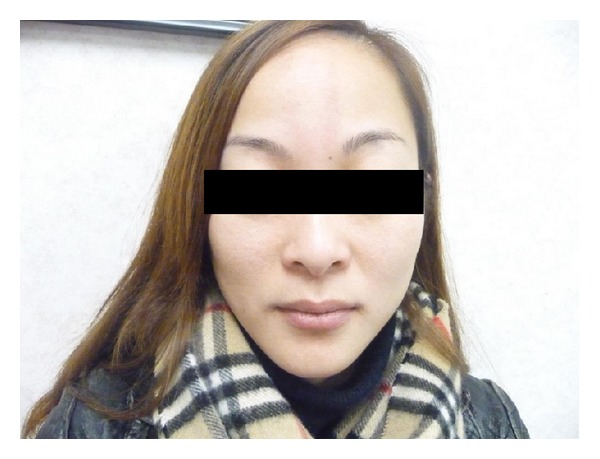
A faint erythematous patch noted on the forehead and glabellum of a 33-year-old patient.

A 33-year-old Chinese female presented with pain in the neck, back, and shoulders as a result of a motor vehicle accident. On examination, there was tenderness and hypertonicity in the paraspinal muscles in the cervical area, paraspinal muscles in the lumbar area, and trapezius muscles. Incidentally, a salmon patch was noted on the forehead and the glabellum (Figure 1). The patch was faintly erythematous in color. There were no other salmon patches noted on any parts of her body. The rest of the physical examination was unremarkable. In particular, she did not have dysmorphic features or hepatomegaly.

According to the patient, the patch was present at birth but was erythematous in color. She did not have similar erythematous patches elsewhere such as the eyelids, nose, philtrum, lips, and the occipital areas. She recalled that the patch would deepen in color with crying and vigorous activity. The color of the patch had become lighter over time. She was born to a gravida 3, para 2, 24-year-old mother at term following a normal vaginal delivery and an uncomplicated pregnancy. The mother was not on any medication or alcohol during the pregnancy. The parents were nonconsanguineous. No family members had similar skin lesions in adulthood.

## 3. Discussion

Clinically, the lesions of salmon patches are scarlet to pink, flat, can be totally blanched, and usually deepen in color with vigorous activity, crying, straining with defecation, breath-holding, or changes in ambient temperature [4]. In white infants, they are usually bright red or pink and are darker in oriental or black infants [4]. The lesions are most commonly found on the nape, followed by the glabella and eyelids [3]. Other less common sites are the nasolabial folds, lips, and sacral area [5]. Salmon patches are usually symmetric, with lesions on both eyelids or on both sides of midline [6]. Prominent lesions in the glabella are associated with Beckwith-Wiedemann syndrome and fetal alcohol syndrome [4, 7]. Salmon patches are generally not associated with extracutaneous anomalies [5]. In spite of their midline location, most salmon patches, except those in the sacral area, are not associated with spinal dysraphism [5].

Salmon patches should be differentiated from port-wine stain (nevus flammeus) and congenital medial frontofacial capillary malformation [8]. Port-wine stain is a capillary malformation characterized clinically by persistent macular erythema and pathologically by ectasia of the papillary and superficial reticular dermal capillaries, which are otherwise lined by normal-appearing flat endothelial cells. The lesions of port-wine stain are usually unilateral and segmental and do not follow the lines of Blaschko. The lesions often become dark-red during adolescence and violaceous with advancing age. Although port-wine stain can occur anywhere on the body, the most common site is the face. The lesions grow with the child and persist throughout life. Although usually an isolated finding, port-wine stain is also a typical feature of Sturge-Weber syndrome and Klippel-Trenaunay syndrome.

Congenital medial frontofacial capillary malformation simulates a salmon patch but differs from a salmon patch in that the lesion is more extensive, extending from the forehead and glabella to the nose, philtrum, and upper lip; the color is more intense; and the lesion fades more slowly or incompletely [8]. Familial cases of congenital medial frontofacial capillary malformations have been reported [9]. Our patient did not have similar erythematous patches elsewhere such as the eyelids, nose, philtrum, and lips at birth. As such, in a strict sense, she did not have a congenital medial frontofacial capillary malformation. Some investigators, however, believe that salmon patches on the forehead and the glabellum may be a forme fruste of congenital medial frontofacial capillary malformation.

Salmon patches tend to fade and disappear with time; nuchal lesions tend to persist longer [3, 10, 11]. Leung and Telmesani examined 808 Caucasian newborn term infants (440 males and 368 females) and 1,575 Caucasian children for the presence of salmon patches [2]. The patches were present in 192 (43.6%) males and 161 (43.8%) females in the neonatal period. The most frequent site was the nape, followed by the glabellum, eyelids, nose, and upper lip. Salmon patches were not detected in boys after age 6 and in girls after age 5. On the other hand, Oster and Nielson detected nuchal salmon patches in 501 (46.2%) of 1,084 Danish school-aged girls and 382 (35.1%) of 1,087 Danish school-aged boys [12]. Corson found nuchal salmon patches in 13 (4.7%) of 275 medical students [10]. Verbov and Steinberg examined 188 hospital inpatients and old-aged residents (67 males and 121 females), aged 60 years and over, for the presence and absence of a salmon patch over the occiput and nape [11]. Forty (60%) of the males and 51 (42%) of the females showed typical nuchal patches. From these studies, it seems clear that salmon patches are common in the neonatal period. These patches, except those located in the occiput and nape, tend to disappear or significantly regress with time. Those in the occiput and nape tend to persist longer. Facial lesions are rare after puberty and have not been reported in adulthood. We herein report the occurrence of a salmon patch on the forehead and glabellum of a Chinese adult. With the reporting of this case, it is hoped that similar cases will be forthcoming. Familiarity of the existence of salmon patch on the forehead and glabellum in adulthood would allow a straight forward diagnosis to be made and unnecessary referrals to be avoided.

The persistent salmon patch on the face of an adult is benign and not associated with any neurocutaneous syndrome or underlying vascular abnormality. The color of the lesion can be ameliorated with laser therapy if cosmesis is a concern.

## 4. Conclusion

Salmon patches, except those located in the occiput and nape, tend to disappear or significantly regress with time. Facial lesions are rare after puberty and have not been reported in adulthood. We report a 33-year-old Chinese female with a salmon patch on the forehead and glabellum. To our knowledge, the occurrence of a salmon patch on the forehead and glabellum in adulthood has not been reported.
